# Association Between the Success of an Invasive Macrophyte, Environmental Variables and Abundance of a Competing Native Macrophyte

**DOI:** 10.3389/fpls.2019.00514

**Published:** 2019-04-25

**Authors:** Mikaela Marques Pulzatto, Eduardo Ribeiro Cunha, Mário Sérgio Dainez-Filho, Sidinei Magela Thomaz

**Affiliations:** ^1^Núcleo de Pesquisas em Limnologia Ictiologia e Aquicultura – Nupélia, Universidade Estadual de Maringá, Maringá, Brazil; ^2^Department of Wildlife and Fisheries Sciences, Texas A&M University, College Station, TX, United States

**Keywords:** invasive species, competition, modeling statistics, standing water, fresh water

## Abstract

The success of invasive species depends on the overcoming of abiotic and biotic filters. Abiotic variables likely have greater relative importance over invasion at broad spatial scales, while biotic interactions are more important at fine spatial scales. In this study, we tested the hypotheses that (i) the abundance of the invasive *Hydrilla verticillata* is more correlated with abiotic factors than with competing native species at broad spatial grain; and that (ii) *H. verticillata* abundance is more correlated with competing native species than with abiotic factors at fine spatial grain. Here, we considered spatial scale as the grain size (*i.e.*, the extent of sampling unit) assuming broad spatial scales as a large area encompassing the entire patches of macrophytes, and fine spatial scales as a small area inside one macrophyte patch. We collected the abundance of hydrilla and the competing native species along with environmental variables in a large subtropical reservoir. To evaluate how the relative importance of the abiotic factors and the competing native species vary between spatial grains we used Bayesian Generalized Linear Models. At broad grain, the abundance of the competing native species, maximum fetch (positive correlation), turbidity and conductivity (negative correlation) were the most important factors to explain the hydrilla abundance. At fine grain, alkalinity, total organic matter of the sediment and the abundance of a competitive native species (all negative correlations) were the most important variables. Our results indicate a greater importance of abiotic factors at broader grains while competitive interactions seem to be important only in the finer spatial grains. Environmental heterogeneity may explain the positive correlation between native and invasive abundances at broad grain, while the negative correlation at fine grain suggests the effect of competition. In synthesis, we show that the abiotic factors that explain the invasion success of a submerged invasive macrophyte are the same in two spatial grains, but the importance of biotic interactions changed with grain. Thus, our data suggest that models that attempt to explain the success of invasive plants, should consider spatial scales.

## Introduction

The success of invasive species in their introduced range depends first on the overcoming of dispersion and establishment barriers (Davis, 2009). These processes are determined by assembly rules, which specify which species of a regional pool can potentially establish in a local pool (Keddy, 1992; Moyle and Light, 1996). Thus, multiple mechanisms are involved at different spatial scales, which are related to the invading potential of the species (traits) and to the susceptibility of invasion of native communities and ecosystems (environmental aspects) (Davis, 2009).

In order to colonize and spread successfully in new areas, invasive species have to tolerate and surpass filters as they pass through the different invasion stages. During the dispersion stage, for example, the potential invader must cross the biogeographic barriers, which occurs through human action (Rahel, 2002). Then, at the establishment stage, the physical and chemical aspects act as physiological filters regulating the invasion process (Rahel, 2002). These filters, which include in aquatic ecosystems the underwater radiation (Barko and Smart, 1981), the chemical characteristics of the sediment (Barko and Smart, 1983; Barko et al., 1991; Wang et al., 2017) and water (Vestergaard and Sand-Jensen, 2000a; Lee et al., 2004) and wave disturbances (Doyle, 2001; Strand and Weisner, 2001), are especially important in the colonization phase of many organisms (Theoharides and Dukes, 2007), especially for invasive aquatic plants. Finally, biotic interactions regulate the invasion success by acting as a biotic filter (Rahel, 2002; Theoharides and Dukes, 2007). The main mechanism associated with the biotic filter is the biotic resistance (Elton, 1958), *i.e.*, the resistance that native species provide to the invasive species, mainly through competition (Gurevitch, 2011; Petruzzella et al., 2018) parasitism and other biotic interactions (Levine et al., 2004).

The roles of abiotic and biotic filters above mentioned vary at different spatial scales (Weiher and Keddy, 1995). The interactions between native and exotic species are considered local processes, and therefore tend to be more apparent and important at fine spatial scale (microcosms or plots of few square meters). Biotic interactions at fine spatial scales have been demonstrated both experimentally (Dukes, 2001) and in observational studies (Case, 1990). In contrast, at broad spatial scales which include sufficient heterogeneity in environmental conditions that do not reflect local processes (e.g. species interactions), the response of invaders tends to be more influenced by abiotic factors (Huston, 1999).

In addition, these environmental filters may also be altered by other impacts resulting from human action, which can make ecosystems more susceptible to invasions (Alpert et al., 2000). This scenario has been widely reported for aquatic environments, where dam construction acts as a modifier of environmental filters (Moyle and Light, 1996; Malmqvist and Rundle, 2002; Rahel, 2002; Johnson et al., 2008). For example, reservoirs alter natural conditions over wide areas, acting as stepping stones for species invasion (Moyle and Light, 1996; Havel et al., 2005; Pitelli et al., 2014). Another typical change relates to sediment trapping, what in turn increases the underwater light availability (Malmqvist and Rundle, 2002; Roberto et al., 2009) and improves habitat suitability for submerged macrophytes. In addition to changing these abiotic filters, the construction of dams can also reduce species diversity and increase instability on biotic interactions (Malmqvist and Rundle, 2002; Havel et al., 2005), decreasing the efficiency of biotic resistance. Owning to these environmental changes, reservoirs favor the establishment of invasive species, including submerged macrophytes.

*Hydrilla verticillata* (L.f.) Royle (Hydrocharitaceae), native to Asia (Zhu et al., 2015) is considered one of the worst aquatic invasive plants worldwide (Cook and Lüönd, 1982; Langeland, 1996; Madeira et al., 2007). It was first recorded outside its native area in 1960 in Florida, United States (Allen, 1976). From then on, *H. verticillata* expanded its distribution to almost all continents, with the exception of Antarctica (Cook and Lüönd, 1982). In 2005, *H. verticillata* was first recorded in the Paraná River basin in Brazil (Sousa, 2011). Since then, its distribution has expanded and it reached several reservoirs (Thomaz et al., 2009; Pitelli et al., 2014). The increase in the water transparency promoted by reservoirs in the Paraná River basin and the propagule pressure from the upstream reservoirs facilitated the invasion in several habitats (Thomaz et al., 2009).

Considering the potential ecological, economic and social impacts of *H. verticillata* and its wide global distribution (Cook and Lüönd, 1982; Langeland, 1996; Sousa, 2011), evaluating the importance of environmental and biotic factors that influence its success becomes a task that interest invasion biologists and environmental managers. In this work, we quantified the relative importance of abiotic variables (abiotic filter) and of a competing native species (biotic filter) on the performance of *H. verticillata* in a subtropical large reservoir. Considering the importance of spatial scales to determine the role of abiotic and biotic variables on species performance (Duarte and Kalff, 1990), we used two databases, one representing broad spatial grain (entire patches of macrophytes) and another at fine spatial grain (small area inside one macrophyte patch), to address our questions.

We tested the hypotheses that (i) *H. verticillata* abundance is more correlated with abiotic factors than with competing native species at broad spatial grain; and that (ii) *H. verticillata* abundance is more correlated with competing native species than with abiotic factors at fine spatial grain. These hypotheses are based on the assumption that abiotic variables tend to be more important for invader establishment in aquatic environments (Moyle and Light, 1996) and that abiotic variables are more important at broad spatial grains while interspecific interactions (biotic filters) are more important at fine spatial grains (Huston, 1999; Fridley et al., 2007). Thus, we expect that the native species *Egeria najas* Planchon (Hydrocharitaceae), which is a strong and potential *H. verticillata* competitor, may hamper the invasive species success at the fine grains. We chose this native species because it belongs to the same family of *H. verticillata* and they are morphologically similar (Sousa, 2011), which may enhance their interactions. *Egeria najas* is also the most important submerged species in terms of occurrence and abundance at the Itaipu Reservoir (S.M. Thomaz, unpublished), what makes it potentially the most important competitor. In addition, the literature shows that a single competitor plant, as the submerged macrophyte *Vallisneria americana* Michx., also of the family Hydrocharitaceae, is capable of effectively reduce establishment of hydrilla fragments (Owens et al., 2008).

## Materials and Methods

**FIGURE 1:**
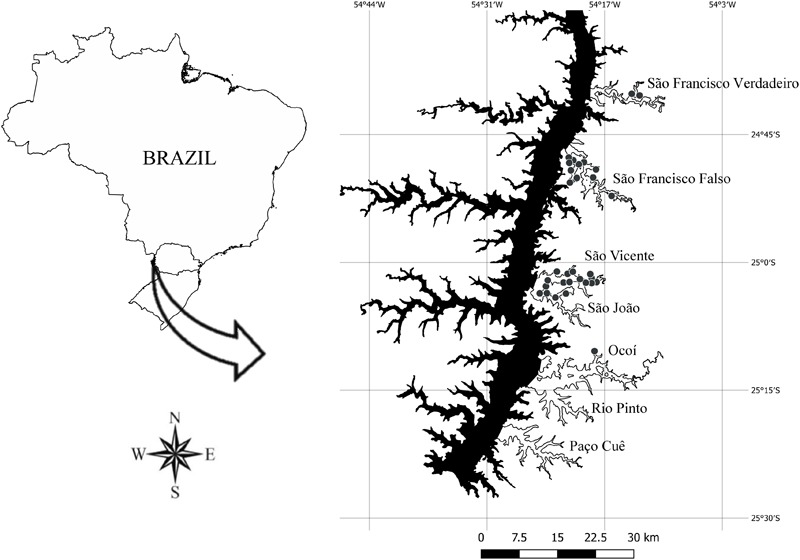
Map of the Itaipu Reservoir, Brazil (Geographic Coordinate System – EPSG: 4326). The sites at broad spatial grain (*n* = 195) are distributed in the lateral arms: São Francisco Verdadeiro (*n* = 28), São Francisco Falso (*n* = 29), São Vicente (*n* = 27), São João (*n* = 29), Ocoi (*n* = 27), Rio Pinto (*n* = 26) and Paço Cuê (*n* = 29). The sites at fine grain (*n* = 31) are represented by grey points on the lateral arms: São Francisco Verdadeiro (*n* = 2), São Francisco Falso (*n* = 11), São João (*n* = 4), São Vicente (*n* = 13) and Ocoi (*n* = 1). The sites sampled are located along the Brazilian margin of the reservoir.

The Itaipu Reservoir (24° 05′- 25° 33′ S, 54° 00′- 54° 37′ W), located between Brazil and Paraguay, was formed by damming the Paraná River in October 1982 (Figure 1). It has an area of 1350 km^2^, an average depth of 22.5 m, a length of 170 km and an average width of 7 km. It has a residence time of about 40 days, being shorter in the main axis of the reservoir (about 29 days). Its hydrometric level is relatively stable (oscillations of less than 1 m per year). The seven arms evaluated in this study differ in relation to the physical and chemical characteristics (Thomaz et al., 2009), varying from oligotrophic to eutrophic conditions.

The large margin development and the shallow areas in the arms favor the growth of aquatic macrophytes in Itaipu. In addition, damming caused the reduction of water velocity, the loss of periodicity and the amount of upstream flood pulses, and the increase of underwater radiation in the entire reservoir due to sediment deposition (Malmqvist and Rundle, 2002). These factors favored the colonization and growth of submerged aquatic macrophytes. Among the 14 submerged species recorded for the area, the most important in terms of occurrence and abundance are those belonging to the family Hydrocharitaceae. Among them, the most frequent ones are the native *E. najas*, found in most arms with high biomass and frequency, and the invasive *H. verticillata*, first recorded in 2007 in Itaipu Reservoir (Thomaz et al., 2009). Other submerged species may occur, but they are historically very rare in the reservoir. According to the monitoring program in Itaipu, *E. najas* and *H. verticillata* reached 89.7% of the total sample effort of all the submerged macrophytes (10 species in total) (authors’ personal data – data collected in 2015–2017). Given the low significance of the other submerged macrophytes as possible competitors for *H. verticillata* and considering their low occurrence in the entire reservoir, we selected only *E. najas* as an indicator of competition.

In terms of spatial scale, we particularly refer to the grain size, which is related to extent of sampling unit (*sensu* Wiens, 1989). We defined the grain at the broad scale as a large area encompassing the entire patches of macrophytes or even more than one patch (a transect c.a. 100 m long). At this grain, the macrophyte patches could be either monospecific or with mixed species. On the other hand, we defined the grain at the fine scale as a small area inside one macrophyte patch (a square with 0.5 m side length). Studies investigating biomass variation at small spatial scales have successfully used similar or even smaller sampled areas (Thomaz et al., 2006; Olsen et al., 2019). In addition, we used a fine spatial grain because we wanted to observe the characteristics of the microhabitat of the macrophytes, i.e., in the case of submerged macrophytes, where they are rooted. Considering that these two species are herbaceous small plants, an area of 0.25 m^2^ is enough to encompass even hundreds of ramets, and then is enough to represent their local abundance. Moreover, such grain extent allowed sampling sites where only a single species occurred and also where both species occurred together (where they potentially compete).

### Samplings at Broad Spatial Grain

In April 2017, abundance data of *H. verticillata* and *E. najas* and environmental variables were collected at 195 geo-referenced sites (Geographic Coordinate System – EPSG: 4326) (Figure 1). These sites were distributed in seven arms of the Itaipu Reservoir (Figure 1), aiming to cover an extensive area with wide variation of environmental conditions. Among the 195 sampling points, *H. verticillata* and *E. najas* co-occurred in 82 points, *H. verticillata* occurred alone in 8 points and *E. najas* occurred alone in 70 points; in the others 35 points, none of them occurred, providing information about potential limiting environmental factors.

In order to determine the relative abundance of each species in each site, a sinuous transect were sampled parallel to the shores of the reservoir (about 80 to 100 m in length, over an area of approximately 3000 to 5000 m^2^). We performed these surveys from a boat at constant low speed. We carried out the samplings up to ca. 4 m depth, which is, in general, the maximum depth of occurrence of the submerged macrophytes in Itaipu (unpublished data). At each transect, we dragged 12 approximately equidistant points with a rake attached to a pipe (4.2 m). In each of these trawls, we measured the abundance rating of each species of macrophyte, which ranged from 0 (absence) to 5 (maximum biomass, with canopies reaching the surface). We estimated the relative abundance for each species of macrophyte per site by adding up all the abundance rating values measured in each transect, to obtain the additive abundance rating. Thus, macrophytes’ additive abundance rating varied potentially from 0 (absence) to 60 (12 points with abundances values of 5) (based on Yin and Kreiling, 2011). This method is highly efficient to represent the relative abundance of submerged macrophytes (Yin and Kreiling, 2011).

At each site, besides the biotic variable (additive abundance rating of *E. najas*), we measured four additional variables representing the abiotic conditions potentially related to the additive abundance rating of *H. verticillata* at broad grain (Table 1). In order to obtain the littoral slope, which is an indication of the extent of the potential area colonized by macrophytes, we used Equation 1:

**Table 1:** Indicators of biotic interaction, disturbances and resource and their respective variables for broad (a) and fine (b) spatial grains.

Indicator	Variable
Competition	*Egeria najas* additive abundance rating^a^ *E. najas* biomass^b^
Wind disturbance	Maximum fetch^ab^ Littoral slope^ab^ † Depth^b^†
Light availability	Turbidity^ab^ Depth^b^†
Carbon availability	Conductivity^a^ Alkalinity^b^
Sediment quality	Littoral slope^ab^† Available nitrogen^b^ Available phosphorus^b^ Organic matter^b^

*^†^The same variable can represent more than one type of indicator.*

(1) $$slope(\%)=\frac{ad}{hd}\times 100$$

Where: *ad* = altitude difference and *hd* = horizontal distance between the altitudes 215-220 m a.s.l. These altitudes correspond to approximate depths where macrophytes colonize the reservoir (data of this work). We obtained the altitudes used to estimate slopes, using satellite imagery.

The maximum fetch represents the maximum open water distance (without crossing the reservoir shore or islands) over which the wind is able to run in a specific direction (Håkansson, 1981; Burrows et al., 2008). Its values were obtained with ArcGIS software (version 9.1), using a reservoir map (Geographic Coordinate System – EPSG: 5880) to measure the maximum distances from the sampling site to the next shore or island. We did not correct the fetch measure for wind velocity, due to the scarcity of wind measurement stations for the large surface area of the Itaipu Reservoir. Even though, we can consider our fetch measure a surrogate of disturbances in macrophyte patches via wave action, as shown previously for the same sampling points by using the same protocol (Thomaz et al., 2003, 2009). We measured the turbidity and the conductivity with a multi-parameter HORIBA sensor. We highlight that the conductivity and alkalinity are highly and positively correlated in the Upper Paraná waters (Roberto et al., 2009) and thus, the former variable can be considered an indicator of inorganic carbon availability, in addition to total ions concentration. Both variables were measured close to macrophyte stands to minimize their effects on local limnological conditions (Carpenter and Lodge, 1986; Duarte and Kalff, 1990).

### Samplings at Fine Spatial Grain

In May 2017, we obtained the relative abundance of each species in each site by measuring the biomass of the two submerged macrophyte species and the environmental variables in 31 geo-referenced sites (Geographic Coordinate System – EPSG: 4326) distributed heterogeneously in five arms of the Itaipu Reservoir (Figure 1). We were looking for a biomass gradient among patches, from the absence of plants to well-established patches of both submerged macrophyte species, and also an environmental gradient. Therefore, considering that *H. verticillata* is widely distributed in the whole reservoir, we rather distributed sampling sites along the environmental gradient than distributing sampling sites homogenously along the reservoir. For this, we haphazardly selected the sampling sites across the arms of the reservoir from a boat, to capture a gradient for all explanatory variables considered. In order to ensured sample independence, we maintained long distances among sampling sites (Figure 1). Among the 31 sampling points, *H. verticillata* and *E. najas* co-occurred in 25 points, while *H. verticillata* occurred alone in one point, *E. najas* occurred alone in three points, and there were only two points where neither occurred.

In 16 sampling points, we collected the biomass of each species by delimiting an area of 0.25 m^2^ with a steel box (1.5 m high × 0.5 m × 0.5 m – quadrat of 0.25 m^2^) and twisting a rake in a 360° turn. After, we collected all the remaining material inside the quadrat with the aid of the rake to remove the plants from the sediment. We carefully washed the plants in tap water to remove excess sediment and attached material and dried them in an oven at 70°C to constant weight. For these quadrats, we obtained the total biomass by adding the biomass of the rake with the biomass remaining in the box. For the other 15 sites, the biomass was obtained only with the rake (without the presence of the box), to facilitate and streamline our samplings. In this case, we predicted the values of biomass per m^2^ through one simple linear regression between the two biomasses obtained at the previous sampling points (from the rake and from the steel box) for each species. The data were transformed (*X*^0.28^, that corresponds to a transformation between cube and fourth root that best linearized data) for both species in order to reach the regression assumptions.

At each site, we collected eight environmental variables potentially related to the biomass of *H. verticillata*, representing the environmental conditions at fine spatial grain (Table 1). All the variables, except those related to sediment, slope and depth, were measured before sampling the plants, near the border of the macrophyte patches, in order to minimize the effects of the plants on the limnological conditions (Carpenter and Lodge, 1986; Duarte and Kalff, 1990). We measured the slope using Equation 1, with *ad* as the depth difference between two measures taken and *hd* as 4.6 m that represents the distance between both ends of the boat where we took the measures. We obtained slope and depth near to the quadrat. We quantified maximum fetch following the same protocol described previously at the broad grain. We quantified the turbidity with a multi-parameter HORIBA sensor. The total alkalinity (a surrogate of inorganic carbon availability) was determined through Gran titration (Carmouze, 1994). We measured both variables close to macrophyte patches by the same reasons presented above (See “*Samplings at broad grain*”). Sediment was collected inside the patches where plants were collected; we used a Petersen grab and frozen the samples for further analyses of available N (Bremmer, 1965) and available P (Stainton et al., 1977). The percentage of sediment organic matter (OM) was obtained by the difference of sediment weight before and after burning sediment samples in a muffle at 550°C for 4 h (Teixeira et al., 1965).

### Data Analysis

In order to assess for independent contribution of environmental variables on the additive abundance rating (at broad grain) and biomass (at fine grain) of the invasive macrophyte *H. verticillata*, we used Bayesian Generalized Linear Models. For obtaining standardized coefficients, we scaled prior distributions of parameters using zero mean priors divided by two standard deviations. For the broad grain, as the additive abundance rating are discrete measures and showed high over-dispersion of the data, the response variable was modeled following a Negative Binomial distribution; the *theta* value for describing the shape of the Negative Binomial distribution was obtained by using maximum likelihood techniques. Before analysis, we transformed the explanatory variables *E. najas* additive abundance rating, turbidity, and maximum fetch into *ln* (x + 1) to linearize the relations. We used non-informative *a priori* distributions for estimating parameters, which means that sampling data mostly influenced the resulting *a posteriori* distribution.

For the fine grain, we adjusted the models using a Gamma distribution that accounts for continuous measurements and positive skewness of residual distributions. We added a small constant (0.01) to the response variables in order to deal with zero values that the Gamma distribution did not handle. Before the analysis, the explanatory variables *E. najas* biomass, turbidity, maximum fetch and sediment OM were transformed into ln (x + 1), to linearize the relations. Similar to previous analysis, we used non-informative a *priori* distributions for estimating parameters.

We employed a model selection procedure using the Akaike Information Criterion (AIC) for both spatial grains. The competing explanatory models that followed the conservative selection criterion (ΔAIC < 2) (Burnham and Anderson, 2002) were obtained from all possible subsets of models (all subsets approach). If more than three competing explanatory models were selected, we used Akaike’s weighted average model to infer the effects of the variables. To compare the relative importance between abiotic factors and the competing native species for each spatial grain, we divided the variables contained in the explanatory models selected using AIC into the abiotic group and competing native species abundance. For each of these two groups, we used the sum of the relative weights (*wi*) of each model containing at least one variable present in the group.

Owning to technical problems, we missed one alkalinity result and, in this case, we applied the missForest non-parametric method to insert missing data, following the protocols of (Stekhoven and Bühlmann, 2012). All analyzes were performed in the R software (R Core Team, 2017), using the MASS, car, arm, missForest, MuMIn and bbmle packages (Venables and Ripley, 2002; Fox and Weisberg, 2011; Stekhoven, 2013; Gelman and Su, 2016; Barton, 2017; Bolker and Development Core Team, 2017).

## Results

### Environmental Features

In general, sampling at both grains ensured a wide gradient for abundance of the competing native species *E. najas* (Tables 2, 3). However, this species did not reach maximum additive abundance rating (60) at any sampling point at the broad spatial grain (Table 2). In the two grains, most of the sites had low littoral slopes (< 10%) and the maximum fetch values presented great variations among sites (ca. 0.2 – 19 km), indicating a broad gradient of wind disturbance. The sampling depths, measured only at the fine grain, were also relatively low (most sites < 2m) (Table 3). Most of the sites had high water transparency, based on the observed low values of turbidity (Tables 2, 3). The conductivity and alkalinity indicated low inorganic C availability at most sites (Tables 2, 3). In the sediment samples (collected only at the fine spatial grain), the nitrogen attained lower values than phosphorus and OM covered a relatively broad gradient, although most values remained below 10% OM (Table 3).

**Table 2:** Descriptive statistics of the abiotic variables and the competing native species collected in seven arms of the Itaipu Reservoir, at broad spatial grain.

Variables (units)	Minimum	Q1	Median	Q3	Maximum	Mean (SD)
*Egeria najas* additive abundance rating	0	1.5	11.0	24.0	48.0	13.5 (12.4)
Littoral slope (%)	1.17	3.46	5.25	7.42	51.92	6.24 (4.87)
Maximum fetch (km)	0.26	1.37	2.14	3.48	18.68	3.10 (3.22)
Turbidity (NTU)	0	0	0.40	0.85	26.30	1.95 (5.09)
Conductivity (μS cm^-1^)	43	56	58	61	148	59 (8.6)

*Q1: first quartile, which means that about 25% of the numbers in the data set lie below Q1; SD: standard deviation; Q3: third quartile, which means that about 75% of the numbers lie below Q3.*

**Table 3:** Descriptive statistics of the abiotic variables and the competing native species collected in five arms of the Itaipu Reservoir at the fine spatial grain.

Variable (units)	Minimum	Q1	Median	Q3	Maximum	Mean (SD)
*Egeria najas* biomass (g DW m^-2^)	0	0.3	8.4	97.5	1012.6	85.6 (193.5)
Littoral slope (%)	0	2.2	6.3	8.7	14.1	5.9 (3.9)
Depth (m)	0.2	1.1	1.8	2.4	4.0	1.8 (0.9)
Maximum fetch (km)	0.26	2.15	4.16	9.99	17.70	5.70 (4.72)
Turbidity (NTU)	0	0.20	1.20	1.85	7.00	1.31 (1.41)
Alkalinity (mEq L^-1^)	318	411	445	464	734	454 (78)
Sediment nitrogen (μg g^-1^)	0.26	11.06	17.60	27.80	65.53	21.54 (15.05)
Sediment phosphorus (μg g^-1^)	13	26	83	119	155	76 (48)
Sediment organic matter (%)	4.1	6.8	8.4	9.6	16.2	8.7 (2.7)

*Q1: first quartile, which means that about 25% of the numbers in the data set lie below Q1; SD: standard deviation; Q3: third quartile, which means that about 75% of the numbers lie below Q3.*

### Response of *H. verticillata* at the Broad Spatial Grain

**FIGURE 2:**
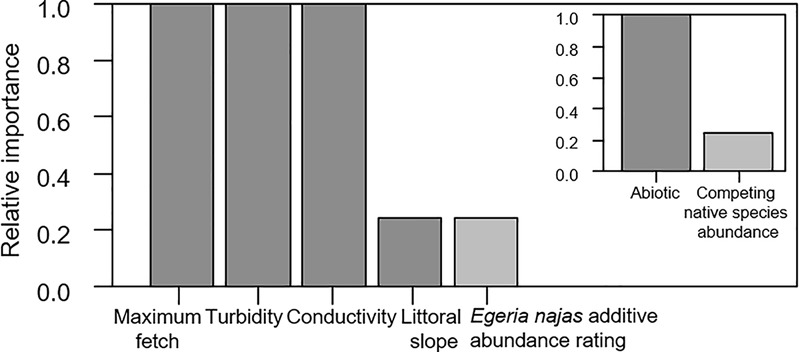
Relative importance of the explanatory variables and of the abiotic and the competing native species *Egeria najas*, based on the weighting of the explanatory models obtained to explain the variability in the additive abundance rating of *Hydrilla verticillata* in the Itaipu Reservoir at broad spatial grain (macrophyte patches).

**FIGURE 3:**
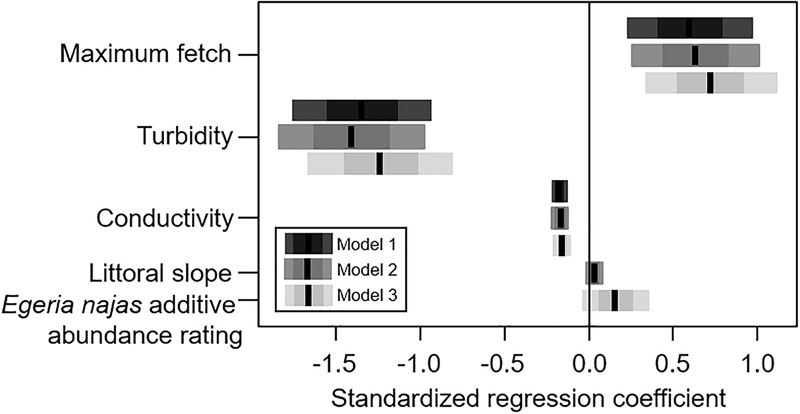
Competitive explanatory models obtained to explain the variability in the additive abundance rating of *Hydrilla verticillata* in the Itaipu Reservoir, at broad spatial grain (macrophyte patches). The black line, the darkest bars and the lightest bars correspond to the mean, the standard deviation and the confidence interval (95%), respectively, for regression coefficients of each of the three competing models.

For the broad spatial grain, despite the low explanatory power of the competing models selected using AIC (R^2^_1_ = 0.176, R^2^_2_ = 0.179, R^2^_3_ = 0.179), consistent statistical effects were observed for all the explanatory variables. In general, the group of abiotic variables presented greater relative importance in relation to the native competing species abundance to explain independent portions of the variability of *H. verticillata* abundance (Figure 2). The maximum fetch, turbidity and conductivity were the most important variables within the abiotic group of variables (Figure 2). Maximum fetch correlated positively with the additive abundance rating of the invasive macrophyte (Figure 3). In contrast, turbidity and conductivity correlated negatively with the additive abundance rating of *H. verticillata* (Figure 3). Only the second and third models selected the slope and the additive abundance rating of the native competitor species *E. najas*, respectively (Figure 3).

### Response of *H. verticillata* at the Fine Spatial Grain

**FIGURE 4:**
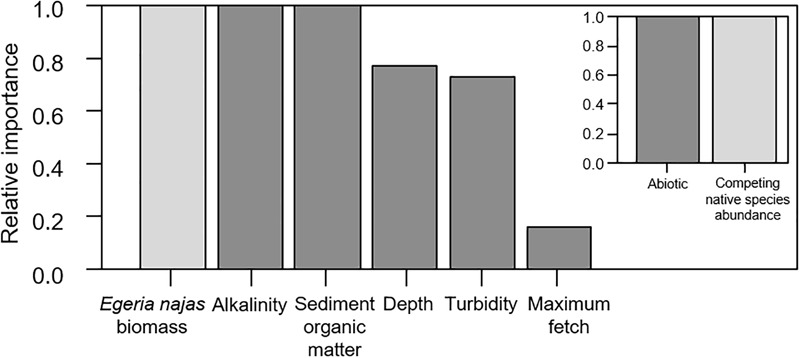
Relative importance of the explanatory variables and of the abiotic and competing native species *Egeria najas*, based on the weighting of the explanatory models obtained to explain the variability in the biomass of *Hydrilla verticillata* in the Itaipu reservoir, at fine spatial grain (quadrat).

**FIGURE 5:**
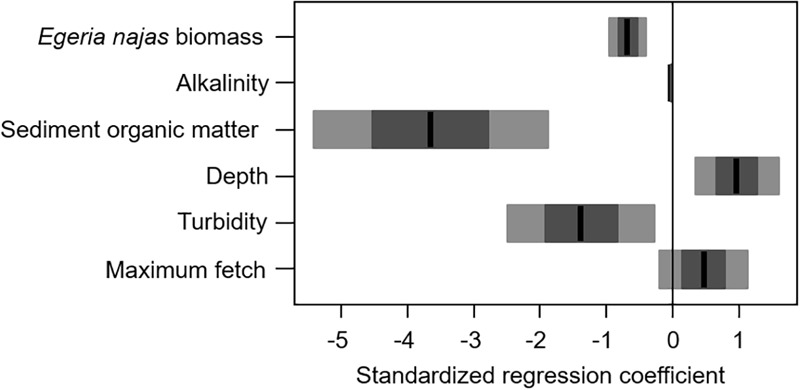
Weighted model for the explanatory models obtained to explain the variability in the biomass of *Hydrilla verticillata* in the Itaipu Reservoir, at fine spatial grain (quadrat). The black line, the darkest bars and the lightest bars correspond to the mean, standard deviation and confidence interval (95%), respectively.

For the fine spatial grain, the weighted model obtained from four selected models had a moderately low explanatory power (R-weighted = 0.38) but presented consistent statistical effects of six of the nine explanatory variables evaluated at this grain. In general, both the group of abiotic variables and the competing native species abundance were equally important to explain independent portions of the abundance variability of the invasive macrophyte *H. verticillata* at fine grain (Figure 4). *Egeria najas* biomass, alkalinity and sediment OM were the variables with the highest relative importance (Figure 4) and they correlated negatively with *H. verticillata* biomass (Figure 5). Alkalinity correlated negatively with *H. verticillata* biomass, paralleling the relation found for conductivity at the broad grain. For the sediment OM, *H. verticillata* tended to occur in sites with low percentage of organic matter, which apparently becomes limiting to macrophyte growth at about 10% (inspection in our graphical analysis; data not shown).

The depth, turbidity, and maximum fetch were less important to explain *H. verticillata* biomass, although they also contributed to explaining independent portions of the invasive biomass variability at the fine spatial grain (Figure 4). The depth correlated positively with the invasive biomass (Figure 5). Both turbidity and maximum fetch paralleled the relationships found for the broad spatial grain, being negatively (the former) and positively (the latter) related with *H. verticillata* biomass (Figure 3, 5). However, at the fine spatial grain, both variables had less relative importance to explain *H. verticillata* biomass than they had at the broad spatial grain to explain *H. verticillata* additive abundance rating (Figure 2, 4).

## Discussion

Our main findings show that at broad spatial grain (i.e., transects), abiotic variables are important to explain the invasive *H. verticillata* abundance, providing support for our first hypothesis. In contrast, at fine spatial grain (quadrats), our findings demonstrate that both the abiotic variables and the native competing species abundance are equally important, which rejects our second hypothesis. These results provide additional support to the idea that environmental factors are more relevant to explain invasive success at broad grain and, at a fine grain, competition may also play an important role (invasion paradox; Fridley et al., 2007).

### Response of *H. verticillata* at the Broad Spatial Grain

Our study is consistent with others that have suggested the greater importance of abiotic factors in relation to biotic factors at broad spatial scales for the success of invasive species (e.g., Stohlgren et al., 2006; Thomaz et al., 2009). Although the definition of scale extension is somewhat subjective (in our study measured as the grain size, sensu Wiens, 1989), broad scales can be understood as those that include environmental heterogeneity high enough so that no single species is able to inhabit the entire spatial grain (Fridley et al., 2007). Indeed, in the wide grain chosen in our work (ca. 3000 to 5000 m^2^), both the invasive *H. verticillata* and the native *E. najas* did not occur in all the sampling points, and they showed a great variation of abundance where they occurred. It shows the importance of the abiotic factors regulating the presence and the abundance of these species in the study area. In additon, broad scales can be understood as those where many individuals can inhabit but with little direct interaction (e.g., competition) among individuals within their neighborhoods (Fridley et al., 2007). Thus, the strength of local competitive interactions decreases while the variation of environmental conditions increases with the scale (Fridley et al., 2007). Therefore, extrinsic factors, rather than biotic interactions, tend to determine the structure of communities at broad scales (Davies et al., 2005), which is consistent with our results.

In this study, *H. verticillata* abundance positively correlated with maximum fetch and negatively correlated with turbidity and conductivity at broad spatial grain. Although the presence and abundance of most macrophyte species tend to be negatively related to fetch, a surrogate of wind disturbance (Doyle, 2001; Thomaz et al., 2003; Azza et al., 2007), some submerged species might be positively affected by waves, when the disturbance is mild (e.g., Doyle, 2001). The positive relationship we found for the abundance of the invasive *H. verticillata* and fetch are in line with previous studies performed in the same study area (Thomaz et al., 2009). The successful performance of *H. verticillata* in sites more exposed to wind is likely related to the presence of well-developed root systems and resistance organs (tubers and turions) that allow a rapid regeneration after disturbances (Langeland, 1996; Bianchini et al., 2010; Sousa, 2011). In addition, wind disturbances can break plant stems and generate propagules, enhancing the propagule pressure. Due to the rapid regeneration of macrophyte fragments (Kuntz et al., 2014; Bickel, 2017), *H. verticillata* is able to rapidly colonize new sites, benefiting from dispersing capabilities enhanced by waves in places with long fetch values.

Light availability has been considered one of the main predictors of submerged macrophyte abundances (Duarte et al., 1986), including *H. verticillata* (Sousa et al., 2009), which explains the negative response of *H. verticillata* to increasing turbidity. We also found a negative relation between abundance of *H. verticillata* and conductivity, which can be likely explained by the effect of ion absorption (including inorganic carbon) by submerged macrophytes. In this respect, it is worth mentioning that macrophytes belonging to the elodeids group (including *H. verticillata*) are able to use HCO_3_^-^ as a carbon source (Madsen and Sand-Jensen, 1991; Vestergaard and Sand-Jensen, 2000b) and may be capable of reducing this ion from the water column. In experiments, for example, the high photosynthetic rates of *H. verticillata* can reduce inorganic C to near zero (Van et al., 1976; S.M. Thomaz, unpublished data from Itaipu Reservoir). The effects of submerged macrophytes on carbon concentration might be enhanced where inorganic carbon is less available, like in Itaipu (alkalinities values usually < 500 m Eq L^-1^; see Table 3). Thus, even that we conducted the sampling near the macrophyte patches, the water mixture probably kept the electrical conductivity low near macrophytes, which likely explains the negative relation between *H. verticillata* abundance and conductivity observed here.

Although others have reported a negative correlation between the littoral slope and macrophyte biomass (Duarte and Kalff, 1986) and macrophyte diversity (Thomaz et al., 2003), we found a positive relationship between littoral slope and *H. verticillata* abundance. It is important to highlight that only one model selected the slope, resulting in a weaker and less consistent relation with the abundance of *H. verticillata*, and so, interpretations should be made with caution. The conflicting result we found here might be associated with several non-mutually exclusive potential causes. For example, the abundance of *H. verticillata* is likely to be smaller in sites with gentle slopes, which are more sensitive to water level fluctuations, which also negatively affects submerged macrophytes through desiccation (Beklioglu et al., 2006; Carmignani and Roy, 2017).

### Response of *H. verticillata* at the Fine Spatial Grain

The effects of environmental heterogeneity and dispersion on invasive success are minimized at fine spatial scales where individuals interact directly (Fridley et al., 2007). Our study pointed an increase of the importance of the native *E. najas* competing species to explain *H. verticillata* abundance at the fine spatial grain. However, different from our expectations, our results also suggested an important role of abiotic factors at the fine grain. This finding is, in some degree, congruent with other studies (e.g., June-Wells et al., 2016). Species competitiveness may change according to environmental variations (Moyle and Light, 1996), highlighting the importance of both factors at fine spatial scales, as we found here.

In this study, the *H. verticillata* abundance correlated negatively with alkalinity and sediment OM at the fine spatial grain. The negative correlation between the invasive macrophyte abundance and alkalinity paralleled the one found between the invasive abundance and conductivity at broad spatial grain, which is not surprising. For the negative relationship found between *H. verticillata* and sediment OM, this outcome may reflect accumulation of phytotoxic compounds from aerobic decomposition that is commonly observed in organic sediments (Barko and Smart, 1983; Wu et al., 2009). In fact, similar patterns were previously observed in other ecosystems in the Upper Paraná River (Sousa et al., 2009).

Depth and maximum fetch correlated positively with *H. verticillata* abundance at the fine spatial grain, although to a lesser relative importance than found at the broad spatial grain. This species shows a particular ability to colonize deep areas (maximum 4 m in this study, 4.0-7.3 m - (Thomaz et al., 2009), where the native species cannot survive (< 2 m for *E. najas*; Thomaz et al., 2003). Colonizing different zones of the aquatic environment allows this invader macrophyte to avoid the high biotic resistance imposed by the higher density of native species at lower depths (Thomaz et al., 2003, 2009), reducing competition between them. In addition, the high abundance of other macrophytes in shallower areas increases the concentration of sediment OM (Godshalk and Wetzel, 1978), which should also indirectly explain why *H. verticillata* was favored in deeper areas. In relation to maximum fetch, the same rationale employed to explain the findings at broad spatial scale may apply at fine spatial scale. In addition, moderate wave action increases water flow and reduces the boundary layer around the leaves, enhancing CO_2_ uptake (Madsen et al., 2001), and reducing periphyton growth (Istvánovics et al., 2008). Both processes increase the photosynthetic rates of submerged macrophytes and probably explain why *H. verticillata* attains higher abundances where fetch is longer.

The negative correlation between turbidity and *H. verticillata* abundance was also similar to results we found at the broad spatial grain. However, the relative importance of this variable was smaller at the fine than at the broad spatial grain. The greater variability of turbidity between the different arms obtained at the broad spatial grain can explain this finding (Tables 2, 3). Thus, light availability may have been more limiting, and therefore its explanatory importance was greater at the broad spatial grain.

### Spatial Grain Dependency of the Importance of Native Competitor on the Invasive Performance

The abundance of the competing native species *E. najas* correlated positively with the invasive *H. verticillata* abundance at broad spatial grain, but the abundance of both species correlated negatively at fine spatial grain. Opposing correlations between indicators of the success of invasive and native species (usually expressed by the diversity of both groups) at different spatial scales have been reported by others (Herben et al., 2004; Davies et al., 2005; Fridley et al., 2007). As the spatial scale (or grain) increases, the variation of resources among local habitats (abiotic heterogeneity), rather than the average of these resources, enhances the native and exotic cumulative diversity, favoring the co-occurrence among them (Davies et al., 2005; in this specific study, the spatial scale varied from a 1 m^2^ quadrat to a 23 716 m^2^ block in a grassland). Then, high co-occurrences between the invasive and the native species would exist at large spatial scales in case of a great spatial heterogeneity (Fridley et al., 2007), which is observed in the arms of the Itaipu Reservoir (Thomaz et al., 2003). A great environmental heterogeneity allows more species (including invasive ones) to co-occur, because there is a great variability of available resources and abiotic conditions to be explored (Davis et al., 2000; Tilman, 2004). Thus, at broad spatial scales, one expects to find positive relations between the performance of invasive species and native ones, as observed for the two species evaluated in this study.

The negative native-invasive relationship we found at fine spatial grain should be more cautiously interpreted, since the abundance of *E. najas* presented less influence on abundance of *H. verticillata*. Even though, this result may open some questions about the mechanisms involved in the interaction between both species. We can suggest that this relationship may be related to factors associated with the niche theory, considering that both species are phylogenetically close and functionally similar (Sousa, 2011). We observed that when both plants were neighbors, the abundance of *H. verticillata* usually decreased with rising *E. najas* abundance. It suggests competition between species may occur, or they differ in terms of environmental optima due to the different physiological tolerances (Fridley et al., 2007). Both observational studies (Sousa et al., 2009) and experimental studies (Thomaz et al., 2012; Silveira et al., 2016) have been describing an indication of inter-specific competition between *H. verticillata* and *E. najas* and differences between their environmental optima. However, stronger conclusions require more studies investigating mechanisms driving this relationship.

## Concluding Remarks

In summary, we found that similar abiotic factors (mainly the fetch and underwater light) might play an important role regulating the success of an invasive submerged macrophyte at both large and fine spatial grains. Particularly at fine grains, other abiotic factors related to sediment fertility and stress, like organic matter, become important determinants of the invasive success. Moreover, in addition to mechanisms driven by abiotic constraints, competing interactions may relate to invasion inhibition only at these fine spatial grains. It is important to note, however, that the explanatory power of the selected models was not strong, and thus the generalizations should be considered with caution. It is worth noting also that the result obtained here are limited to restricted spatial scales in a large reservoir and, so, extrapolation to different spatial scales and other ecosystems (e.g., streams or rivers) deserves additional investigation. Despite of limitations, our major finding suggests that models that attempt to explain the success of invasive plants, should consider spatial scales. Our findings may guide future investigations addressing mechanisms that are more specific regulating invasion success by *H. verticillata* at different spatial scales.

## Author Contributions

MP, MD-F, and ST conceived the ideas and the sampling design. MP and MD-F conducted the field samples. MP and EC conducted the statistical analysis and their interpretations. MP and ST lead the manuscript writing. All authors contributed crucially to the final version of the manuscript and agree with it.

## Conflict of Interest Statement

The authors declare that the research was conducted in the absence of any commercial or financial relationships that could be construed as a potential conflict of interest. The handling Editor is currently organizing a Research Topic with one of the authors ST, and confirms the absence of any other collaboration.
